# Accessing hepatitis C direct acting antivirals among people living with hepatitis C: a qualitative study

**DOI:** 10.1186/s12939-023-01924-4

**Published:** 2023-06-06

**Authors:** Tony Antoniou, Cheryl Pritlove, Dana Shearer, Mina Tadrous, Hemant Shah, Tara Gomes

**Affiliations:** 1 Li Ka Shing Knowledge Institute, Unity Health, Toronto, ON Canada; 2 Department of Family and Community Medicine, Unity Health, Toronto, ON Canada; 3 Department of Family and Community Medicine, University of Toronto, Toronto, ON Canada; 4 Dalla Lana School of Public Health, University of Toronto, Toronto, ON Canada; 5 Leslie Dan Faculty of Pharmacy, University of Toronto, Toronto, ON Canada; 6 Women’s College Research Institute, Toronto, ON Canada; 7 Toronto Centre for Liver Disease, University Health Network, Toronto, ON Canada; 8 Department of Medicine, University of Toronto, Toronto, ON Canada; 9 Institute for Health Policy, Management and Evaluation, University of Toronto, Toronto, ON Canada

**Keywords:** Direct acting antivirals, Hepatitis C, People who inject drugs, Qualitative research

## Abstract

**Background:**

Hepatitis C is curable with direct-acting antivirals (DAAs). However, treatment uptake remains low among marginalized populations such as people who inject drugs. We sought to understand challenges to treatment uptake with DAAs among people living with hepatitis C and compare treatment experiences between people who do and do not inject prescription and/or unregulated drugs.

**Methods:**

We conducted a qualitative study using focus groups with 23 adults aged 18 years and over who completed DAA treatment or were about to begin such treatment at the time of the study. Participants were recruited from hepatitis C treatment clinics across Toronto, Ontario. We drew upon stigma theory to interpret participants’ accounts.

**Results:**

Following analysis and interpretation, we generated five theoretically-informed themes characterizing the experiences of individuals accessing DAAs: “being ‘worthy’ of the cure”, “spatially enacted stigma”, “countering social and structural vulnerability: the importance of peers”, “identity disruption and contagion: attaining a ‘social cure’” and “challenging stigma with population-based screening”. Overall, our findings suggest that structural stigma generated and reproduced through healthcare encounters limits access to DAAs among people who inject drugs. Peer-based programs and population-based screening were proposed by participants as mechanisms for countering stigma within health care settings and ‘normalizing’ hepatitis C among the general population.

**Conclusions:**

Despite the availability of curative therapies, access to such treatment for people who inject drugs is limited by stigma enacted in and structured within healthcare encounters. Developing novel, low-threshold delivery programs that remove power differentials and attend to the social and structural determinants of health and reinfection are needed to facilitate further scale up of DAAs and support the goal of eradicating hepatitis C as a public health threat.

## Introduction

Chronic hepatitis C viral (HCV) infection is a significant global public health concern, affecting an estimated 71 million people worldwide [1]. Because of multiple intersecting social and structural vulnerabilities, including criminalization, poverty and stigma, people who inject drugs remain especially vulnerable to HCV infection and reinfection, [2–7] with an estimated 6.1 million individuals who inject drugs worldwide living with hepatitis C [8]. In Canada, an estimated 194,500 individuals were living with hepatitis C in 2017, [9] with individuals born between the years of 1945 and 1975 and younger individuals who inject drugs comprising the majority of long-standing and incident infections, respectively [10]. Similar to global trends, people who inject drugs are disproportionately impacted by hepatitis C in Canada, with 60–85% of new infections occurring in this population between 2000 and 2016 [11] Further, it is estimated that the prevalence of chronic hepatitis C among people who inject drugs in Canada was 50.7% as of 2017 [12]. Untreated, chronic hepatitis C is a progressive disease associated with diminished quality of life for affected individuals and a substantial burden to health care systems tasked with managing complications such as liver failure and hepatocellular carcinoma [13, 14].

Fortunately, chronic hepatitis C is now curable in almost every infected person with direct-acting antivirals (DAAs) [15, 16]. Unlike older interferon-based regimens, DAAs require shorter courses of therapy, are not administered by injection and are relatively well tolerated [15, 16]. In addition to reducing the risks of end-stage liver disease, liver cancer and death, being cured of hepatitis C improves patient quality of life and extrahepatic manifestations of the disease, including insulin resistance and heart disease [17–21]. Moreover, curing hepatitis C imparts public health benefits by preventing onward transmission and lowering the population burden of disease [22–24]. Consequently, ensuring comprehensive and equitable access to DAAs for all people living with hepatitis C is a necessary prerequisite to the World Health Organization’s goal of eliminating this condition as a major public health threat by 2030 [25, 26].

Yet, despite the individual and public health benefits of curative treatment with DAAs, uptake of treatment remains low in Canada, particularly among people who inject drugs [5, 27, 28]. Specifically, a study of HCV-diagnosed people who inject drugs conducted between 2017 and 2019 found that only 10.6% of individuals had ever received treatment, with only 3.8% receiving treatment at the time of the survey [5]. In addition, a 2018 population-based study found that 39.8% of people diagnosed with hepatitis C who inject drugs had accessed treatment, compared with 48.3% and 60.4% of people with a prior history of injecting drugs and individuals with no such history, respectively [28]. Research examining the experiences of DAA access and treatment among individuals who inject drugs is therefore needed to understand the social relations and broader discourses which limit treatment uptake and to inform the planning and delivery of treatment services for this population. Although past qualitative studies of people who inject drugs have identified potential deterrents to hepatitis C care, including risks of treatment while otherwise asymptomatic, competing priorities and stigma, few of these studies were conducted in the contemporary period of DAA availability where disease-based criteria for accessing drugs have been relaxed to facilitate treatment access [29–33]. Moreover, qualitative research which contextualizes and explains hepatitis C treatment disparities identified in population-based studies of people who do and do not use drugs is lacking. Such research is necessary to compare treatment experiences between these groups and shed light on the mechanisms underpinning treatment disparities observed in population-based studies. This is especially important in a setting such as Canada, where all citizens have publicly funded health insurance and approximately 88% of individuals undergoing DAA therapy have had treatment costs covered by the public health system [34]. Accordingly, we undertook a qualitative study that sought to characterize the perceptions and experiences of hepatitis C treatment among people who do and do not inject prescription and/or unregulated drugs in a large urban centre where access to treatment was not otherwise limited by the availability of treatment specialists and programs to support people who inject drugs. We further aimed to situate these experiences within the broader social relations and discourses in which they are embedded.

## Methods

### Study setting and context

Our study was conducted in Toronto, Ontario, Canada. Ontario is Canada’s most populous province and is home to over 40% of Canadians living with hepatitis C [35]. Although access to DAAs was initially restricted in Ontario to individuals meeting specific thresholds of liver fibrosis, all disease-based criteria for DAA treatment for chronic HCV infection were removed in Ontario in 2017, [36] with all commonly prescribed drugs subsequently added to the provincial drug formulary. At no time was access to DAAs contingent on sobriety or the absence of drug use. Treatment with DAAs is provided principally by specialists and primary care physicians. In addition, several ‘hepatitis C teams’ have been established in Ontario to provide marginalized populations, including people who inject drugs, access to DAAs and additional support to facilitate successful completion of therapy [37].

### Sampling and recruitment

In qualitative research, sampling is not conducted for the purpose of probabilistic generalizability to a larger population [38]. Instead, the goal of sampling is to purposively identify participants who can provide rich, in-depth and detailed information about the phenomenon under study. Accordingly, following ethics approval, we purposively recruited adults aged 18 years and over who were diagnosed with hepatitis C and who had completed DAA treatment or were about to begin such treatment at the time of the study [38]. We partnered with an outpatient specialist-led liver clinic affiliated with a large teaching hospital and three primary-care based hepatitis C clinics to assist us in recruiting a diverse sample of participants according to sex, age and current or past history of injection drug use. Recruitment posters containing study information and contact details were shared with each participating site. We also disseminated study recruitment materials through the Ontario Drug Policy Research Network social media channels and website. Lastly, we employed snowball sampling, whereby study participants inform other prospective participants about the study [38]. Interested participants followed up with a designated contact person at the specialist liver clinic or the research team to confirm eligibility and availability for a scheduled focus group. Participants were compensated with a $50 honorarium.

### Data generation

We conducted four focus groups with 23 adults aged 18 years and over. Because we were concerned that people who inject drugs could experience inadvertent harm or stigma in groups that included people who did not inject drugs, two focus groups were comprised exclusively of participants with a current or past history of injection drug use. The remaining focus groups were comprised of individuals who did not inject drugs, although a small number reported a past history of injection drug use. Although the optimum number of focus groups for research stratified by specific participant characteristics is unknown, a minimum of two focus groups per stratum with ongoing assessment of response saturation is considered sufficient [39, 40]. All participants provided written informed consent and completed a brief sociodemographic questionnaire prior to participating in the focus group. All focus groups were led by an experienced qualitative methodologist (CP) in private rooms located within St. Michael’s Hospital or a private space within the specialist liver clinic. A second member of the research team (TA) attended each focus group to generate field notes and co-lead the session. Prior to commencing the focus groups, participants were briefed about the public nature of focus group activities and the need for confidentiality, and were provided with a general overview of the nature of the topics to be discussed during the session.

We developed a semi-structured focus group guide to elicit participants’ hepatitis C related diagnostic and therapeutic experiences and to understand how they became aware of and accessed DAA therapy. Probing questions captured participants’ perspectives regarding the social and structural factors that promote and hinder access to DAAs and the delivery of hepatitis C related care. While we used the interview guide to address key areas of interest and to stimulate discussion, we allowed participants to steer the discussion toward aspects of their experience that they felt to be most relevant and appropriate. Focus groups lasted between 96 and 145 min and were audio taped and professionally transcribed, with transcripts undergoing a quality check to ensure accuracy.

### Data analysis

Immediately following each focus group, two researchers (CP and TA) debriefed to reflect on the nature of topics discussed, the tone of the group interaction, identify notable exchanges among participants, and compare and contrast emerging insights among the different focus groups. Debriefing notes were summarized with field notes as memos and together with the transcripts, comprised the data corpus available for subsequent analysis and interpretation.

We used constructionist grounded theory to analyze our data [41]. As a first step, we used line-by-line coding of transcripts to construct preliminary codes derived from the words of the participants (e.g., “being responsible”, “brand new life”). Coding is part of an ongoing and iterative process of active engagement with data whereby researchers construct and apply labels to short segments of the data that will ultimately be refined and developed through memo-writing, constant comparison and subsequent rounds of coding into interrelated concepts that provide insight into the phenomenon under study. For each section of coded data, we produced memos that were cross-referenced by transcript, page and line numbers. Memos were written to elaborate on line-by-line coding and to undertake an iterative process of constant comparison to illuminate differences and similarities between and within groups [41]. Using word processing software, similarly coded data were extracted from transcripts and re-assembled as preliminary themes representing how HCV treatment was experienced and perceived by participants. Next, we undertook a process of theoretical coding, [41] interrogating participant accounts, groups of codes and memos using questions such as “What narratives are reproduced and/or resisted?”, “What are the circumstances that produced this interaction?” and “What are participants doing in this segment of data?” In keeping with a constructionist grounded theory approach to data analysis and interpretation, we did not immediately begin our data analysis from a specific theoretical position. However, we were sensitized to Erving Goffman’s work on felt and enacted stigma and Link and Phelan’s model highlighting the role of power in perpetuating stigma through labelling, stereotyping and discrimination, and used these frameworks to support later stages of theoretical coding [42, 43]. Consequently, rather than serving as the foundation for our study, we employed theory primarily as an analytic device for refining and conceptualizing our data following an inductive, data-driven process of generating initial categories. This approach is consistent with the multiple ‘guises’ of theory in qualitative research, in that theory may enter a qualitative research study at various stages, including acting as the underlying rationale for the study and being brought in to a study to support analysis and interpretation [44]. We repeated the process of coding and memo writing, cycling iteratively between the focus group data and theoretical frameworks until we had developed well-theorized concepts that related the accounts of the participants with the objective social relations and discourses in which they were embedded. In this manner, we produced an analysis that was theoretically informed but always grounded in the data.

### Ethical considerations

We obtained written informed consent from all participants. This study was approved by the Research Ethics Board of St. Michael’s Hospital (REB# 18–142).

## Results

### Participant characteristics

Overall, the median age of study participants was 57 years (interquartile range [IQR]: 43, 64), and most (18/23; 78.2%) were born in Canada. Participants who did not use drugs were older (median 65 years; IQR 61.5 to 66.5) than those who did use drugs (median 43 years; IQR 35 to 48). Similarly, years since diagnosis was longer among participants who did not use drugs (median 4.5 years; IQR 2.5 to 17) relative to participants who did use drugs (median 2 years; IQR 0 to 11).

### Findings

We generated five concepts characterizing the experiences of individuals accessing HCV treatment: “being ‘worthy’ of the cure”, “spatially enacted stigma”, “countering social and structural vulnerability: the importance of peers”, “identity disruption and contagion: attaining a ‘social cure’” and “challenging stigma with population-based screening”.

### Being ‘worthy’ of the cure

Access to hepatitis C treatment is an obvious prerequisite to becoming cured and achieving elimination of the disease. However, the accounts of participants describe vastly differing experiences in their ability to secure such access, with participants who did not use drugs describing a fairly uncomplicated pathway to curative care. Conversely, participants who did use drugs and/or were perceived by healthcare providers to have a substance use disorder described a haphazard process that reproduced the felt stigma of the ‘irresponsible drug user’ inculcated through previous encounters with the health care system.

For participants who did not use drugs, access to curative therapy was described as a logical extension of their diagnosis, with no qualifying conditions for care.

P: My family doctor told me, as soon as he said there was that diagnosis, and he said ‘There’s a cure’. To me, that was (laugh) all I wanted to hear. And of course, he referred me to the specialist. And, the rest is, is history, I guess, as it were. So, when I got the diagnosis, I was, he told me there was a cure for it. There was treatment. Um, I referred to Dr. Google, (laugh) for more information, till I came here. And so to me, there wasn’t, and there isn’t a stigma in my case, only because I don’t tell anybody. I don’t feel any urge to tell, except my wife, obviously. But, beyond that, I feel no compunction.

Moreover, once in care, participants who did not use drugs described the various measures taken by clinic staff to ensure an organized treatment experience, from providing the necessary requisitions for bloodwork, assistance with drug coverage, and phone ‘check-ins’ as reassurance and reminders of upcoming appointments. Importantly, these individuals described being ‘made comfortable’ through the orderly process and interactions with the clinic staff that dispelled perceptions of stigma around a diagnosis of hepatitis C.

P: Yeah. I know. (name), and when I first, I came here, Dr. (name) and everybody gave me their hands. That was a good sign that they didn’t afraid of my disease. Of course, they knew I got um, hep C. They had my profile. And that was a good sign, for me. I was calm, calm calmer. And I think what felt great, Dr. (name) did two or three blood tests; they confirmed that I have hep C. They did liver scan. And, they were so informative.

In contrast, participants who used drugs framed treatment with DAAs as an exception rather than an extension of their diagnosis. Specifically, these individuals recounted missed opportunities to be referred to or informed of curative therapy despite abundant interactions with health care providers who were aware of their diagnosis.

P: But, they didn’t once, when I left the hospital, did they ever tell me where to go, for treatment.

P: That’s right, yeah.

P: Never, not once. Not once did they say to me, ‘Here’s where you can go, for possible treatment for your hep C.‘ Not once, in any hospital I was in.

I: Right.

P: Lack of education.

P: They just keep telling me I have it. But I just told them I had it, cause I write it down on that form. So they’d reconfirm to me, what I already told them.

P: Yeah. Back and forth, back and forth.

P: But they never steered me to a place where I could actually get help. And that’s the truth.

According to participants, the lack of information from health care providers regarding curative therapies has fostered an environment where the belief that treatment for hepatitis C entails a protracted course of therapy with interferon persists, deterring people who use drugs and alcohol from actively seeking help. In the absence of referrals and information from health care providers, participants recounted the importance of peers as their gateway to learning about and accessing modern curative therapy.

P: Yeah, I found out through peers, and like -.

P: Word of mouth.

P: I go to the injection site as well, and like, and then, we’d all be talking, we became friends with, so we just, yeah.

P: Kind of like word of mouth.

P: Just like, word of mouth, yeah.

P: Just like how they found out from this. Like, you know what I mean, word of mouth.

P: Mmm-hmm.

P: So I wish there was better information out there –.

However, even after becoming aware of treatment and securing referrals to specialists, participants recounted how access to treatment was not straightforward. Instead, individuals described being subjected to various practices that reinforced felt and internalized stigma of people who use drugs as being ‘irresponsible’, ‘chaotic’ and ‘unreliable’ that had been instilled within them through prior encounters with the health care system. One way these perceptions were reproduced was through interactions with health care providers in which conditions were placed on the receipt of curative therapy. In this manner, participants recounted having to demonstrate their ‘worthiness’ for treatment by first abstaining from drugs or alcohol or providing evidence of ‘stability’ in their lives.

P: Okay? And I was going to try and get treatment. And the doctor said, ‘Unless you quit drinking alcohol, we’re not going to waste - ' they’re not going waste their resources on me. This is when I first started out. So every time I went to see them, he said ‘Have you been drinking?‘ And I said ‘Well,’, ‘No, we can’t do you.‘ So they tried to make me quit for so long, before they would start me on the liver treatment. And I said ‘Well, that’s going to be impossible. Because I’m an alcoholic. I’ve been drinking for forty years.‘ So they wouldn’t treat me for my hepatitis because of my alcoholism. But yet, I was on a methadone program for fourteen years. So, I wanted to get rid of my hepatitis C, but because I couldn’t quit drinking, I didn’t qualify. Cause that’s the way it was, eh, (name), when I first started -

P: Yeah.

P: You had to prove yourself, that you were kind of worthy of –

P: Worthy. Yeah.

The notion of proving oneself ‘worthy’ for treatment was intimately tied to the production of DAAs as costly interventions subject to rationing among those individuals who could satisfactorily ‘qualify’ for treatment. It was not uncommon for individuals to perceive being “looked down upon” while being told the costs of treatment and how, accordingly, they had “one chance” at the cure contingent on proving themselves reliable and deserving of the resources that would be expended for a course of DAAs.

P: ‘You got one chance at this and one chance only.‘ But she said, even before I started, ‘I’m making sure that you’re making a commitment to us. Cause, it’s going to benefit you, right?‘

P: You’re wasting the -.

P: you’re wasting money, and you’re fucked. Like, you -.

P: Yeah.

P: And you won’t get it again.

The acts of placing conditions on curative therapy and re/producing a discourse of deservedness were deterrents to care for participants who use drugs, recounting how they “just gave up” on pursuing treatment and resigning themselves to “dying with hep C”. Participants contrasted these experiences with the care they subsequently received at clinics integrating peer-led treatment programs and harm reduction services with HCV care, where accessing treatment was not situated within narratives and practices that reproduced feelings of worthlessness among clients.

P: She was concerned about me and my hepatitis C. She didn’t care about, you know, what I did to self-medicate.

### Spatially enacted stigma

Participants who use drugs also recounted how seeking care in settings known to provide services for individuals who use drugs can undermine efforts at achieving disease eradication among vulnerable populations by deterring engagement with these sites. Specifically, while participants who did not use drugs accessed specialty services in an otherwise non-descript building lacking in physical markings identifying it as a space offering treatment for hepatitis C, those who did use drugs highlighted the potential for inadvertent disclosure of drug use and hepatitis C status if they are seen entering settings known for the provision of services catering to individuals living with this condition.

P: Even walking in the building, if they see you walking around that area, that building, we know what that building’s about.

I: Right.

P: Yeah.

P: We know what that building’s about. That’s harm reduction. That’s drug addicts. That’s people that are infected there.

In addition, participants who use drugs identified how stigma can be enacted within health care settings by physical layouts clearly demarcating spaces as being for those with hepatitis C.

P: And then see my friend waiting there to see their doctor, and then they’re going to see me going into the hep C nurse. (laugh)

P: Yeah. (laugh)

P: It says right there [on the nurse’s door].

P: It’s like putting a label right on your forehead.

Importantly, participants described themselves as being unable to effect change when potentially problematic signage or layouts were identified to staff. These interactions are exemplars of Link and Phelan’s notion that power differentials can inadvertently produce conditions in which labelling can occur, with the end result being a propagation of stigma that affected individuals are powerless to counter [43].

P: It’s in the waiting room, the first door, and it says ‘Hep C’.

P: Has she changed it yet? (laugh)

P: Yeah, I asked her to change it. She just said ‘No, it’s policy.‘

### Countering social and structural vulnerability: the importance of peers

Central to the accounts of conditional access and establishing one’s ‘worthiness’ for treatment is a neo-liberal discourse emphasizing health as an individual responsibility and substance use as a personal choice, thereby constructing people who use drugs as lacking in self-control and without a sense of collective responsibility for interrupting the transmission of HCV [45–49]. This discourse was reproduced by participants who did and did not use drugs.

P: You know, a lot of people just don’t. And make them aware, ‘Listen, you are responsible. We’re all responsible.‘ And I think that’s a little bit heavy message, but it, in the same way, it is a message, even for the people who are under the influence of, you know, that they are aware of that you are also very responsible. ‘Okay, you made a choice. You’re going to live that kind of a life. But at least be responsible so that you do not transfer the disease.‘ It could be the hep C. It could be the, ah, any other disease as well. It’s not just hep C. Right? So, yeah, I think if we all think of it that way, a little bit of responsibility starts from me and you and you and you. And somehow, if there is a way of letting the people know, over there, who are homeless: it’s their responsibility too.

However, the emphasis on individual responsibility was also challenged by participants who did and did not use drugs who felt that such perceptions ignored the structural forces underlying substance use and which influence the health of people who use drugs, including housing instability, criminalization of drug use and past traumas [2–7, 47].

P: Yeah. You see, here’s where that logic is flawed. I worked with the parole system for years. And I got, I had to read a lot of case files of these people. And a lot of these people who are in the prison system, they’ve been marginalized, either socioeconomically, racially, and they’re, plus there’s the fact that a lot of them are emotionally, sexually and physically abused. I think that of the people that I had to deal with, the inmates I had to deal with, I think eighty percent of them are physically, mentally or sexually abused as children –

P: That’s right.

P: by a caregiver. They all had demons to deal with. Nobody was there to help them. Nobody was there to help them. They end up on the street as kids, cause there’s nowhere else for them to go. And what do they do? Where do they get their warm hug? They get it from heroin.

Consequently, participants who use drugs recounted the importance of integrating hepatitis C treatment with programming and support services required to mitigate their structural vulnerability to adverse health outcomes and optimize the likelihood of achieving a cure. In the absence of such services, therapy was perceived as futile given the need to attend to competing priorities, lack of a means to attend clinic appointments and limited access to safe injection supplies to prevent reinfection. Examples of supports deemed necessary by participants included on-site harm reduction services with hours of operation that align with those during which drug use occurs, financial support to offset the costs of attending clinic appointments, and assistance with food and housing insecurity. Participants stressed the fundamental necessity of involving people who use drugs and people living with HCV in the design and delivery of treatment and supportive programs to ensure that services were rooted in the tacit expertise of people with lived experience as opposed to the knowledge of “someone that’s just read it from a book”. Because of a shared sense of identity, participants explained how they would be more honest with and trusting of peers relative to other health care providers, and how receiving treatment from peers provided a counterpoint to the discrimination experienced in past encounters with the health care system.

P: People would go ‘Wow, I’m not just grabbing a bag and getting looked at dirty.’ I’m getting treated by someone that uses, that’s actually willing to help me.

In addition to involving peers in the provision of material supports and treatment, accounts of participants who use drugs were replete with references to the importance of peer support groups in optimizing access to curative therapies and successfully completing treatment programs. Specifically, peer support groups were described in terms of “having a place to go” and “being in the same boat”, generating a sense of being at ease and acceptance that stood in contrast to past experiences of proving one’s worth for treatment. Furthermore, participants recounted how the act of providing and receiving peer support served as an antidote to the stigma and shame structured within spaces initially perceived as potentially unsafe.

P: But once you’re in the group, you feel like you, we’re all together sort of.

P: Yeah, exactly.

I: Okay.

P: Um, I don’t care that people see me go through (name) door anymore.

P: No.

P: Me neither.

In addition to serving as sources of camaraderie and care that is perceived as non-judgemental, peer-led treatment programs and groups provided opportunities for personal transformation through employment as peer workers and individuals with expertise in the science and treatment of hepatitis C. In this way, some participants became galvanized to counter systemic deficiencies in the lack of access to curative therapies among people who use drugs.

P: All right. Yeah. It’s been, ah, it’s been eye opening, educational, you know, everything. I brought a ton of people through that group now. I’ve worked with that group; I work for that group. So, yeah. It’s life changing, going through those groups.

Participants who use drugs also described how involvement in peer-led activities provided the impetus for larger mobilization efforts aimed at removing disease-based qualifying criteria for DAAs imposed at the time these therapies were first made available and ensuring that the harm reduction services required to prevent reinfection and support safe drug use while undergoing HCV treatment were in place. In this manner, participants seek to challenge upstream determinants shaping access to care and treatment success among people who use drugs.

P: Right? Like, there’s a lot of good people out there fighting, like, (cites names) and all them, right? They’re pushing through it and trying to get big change in the system. Like, we’ve gone through marches downtown Toronto, down to the Ministry of Health and asked for certain criterias to be changed and stuff, but you know? And because society looks at it as, you know, ‘You have hep C. How’d you get hep C? You’re a drug user. Shame on you.‘ Now, they have safe injection sites, which are, you know, they’ll stop quite a bit of the hep C, right? Because the safe injection site now has the tools for people that transmit those – so hopefully, they get the word out.

The need for ancillary services and peer support was less explicitly evident in the narratives of participants who did not use drugs, who typically shared information regarding their diagnosis with close family only. However, as noted earlier, these participants described interactions with clinic staff as caring and non-stigmatizing, such that a need for additional support beyond that received by the treating physicians and nurses was not required.

P: Just everything. Because, like, I felt like a human, treated like a human being.

Although participants who did not use drugs did not express a need for services addressing the stigma and structural causes underlying the acquisition of hepatitis C, these participants recounted their appreciation for a streamlined treatment experience wherein all necessary paperwork and approvals were completed with the assistance of clinic staff and the availability of allied health support staff (e.g., pharmacists) ensured uninterrupted access to treatment and information. For these participants, delivering these efficiencies within a climate of compassion was key to ensuring treatment success.

P: It’s like one-stop shopping, isn’t’ it? (laugh) You come here, and everything gets done for you.

### Identity disruption and contagion: attaining a ‘social cure’

Participants who did and did not use drugs expressed a range of concerns when learning of their diagnosis with hepatitis C, such as feeling shame, being ostracized by others and losing employment. In many cases, these concerns were rooted within a discourse of HCV as being especially contagious and readily transmittable through routine daily practices [7, 48]. Importantly, this perception was internalized and reproduced by participants through referring to hepatitis C as a “dirty, dirty disease” and individuals living with the condition as “being Typhoid Mary”. Accordingly, participants described a disruption in what Goffman referred to as their felt identities, [42] constructing them as being perilous to others and enacted through the adoption of sanitization measures intended to protect family members from acquiring the virus through means not associated with HCV acquisition.

P: Not letting anybody use your knife, your fork, your plates, your spoons, your cups -.

P: Oh yeah, yeah.

P: Right? You can’t let them wear your sweaters, your clothes, your socks.

Moreover, participants described ignorance about the transmissibility of hepatitis C among their social networks, resulting in actions that reinforced the notion of the ‘dirty’ individual who is a vector of illness and amplified feelings of shame and fears of abandonment.

P: ‘You’re sick. You’re – get away from me. Don’t smoke a cigarette of mine. Don’t touch - ‘

P: ‘my fucking plate.‘ You know what I mean?

P: ‘Don’t even come close to me.‘

P: Yeah.

P: Yeah. ‘Don’t come close to me. Don’t kiss me. Don’t hug me.‘

Because of fears of ostracization and transmission to others, it was not uncommon for participants to frame the cure in terms of both viral clearance and the opportunities to ‘cure’ oneself of the problematic imputations associated with the virus. For participants who did not use drugs in particular, a successful cure was defined in terms of a return to a time when life was ‘normal’ and unencumbered by the weight of being a potential source of infection to others and the various forms of stigma associated with this condition.

P: I’ve been through it. I didn’t like it, but now I’m cured. I don’t have to worry about infecting my friends’ children, or my friends, or anybody around me. I don’t have to worry that I’m a walking disease.

In contrast, such restitution appeared out of reach for participants who use drugs. For these individuals, a virologic cure does not automatically translate into a successful ‘social cure’, with claims of being cured being challenged in interactions with health care providers. Consequently, people who inject drugs resign themselves to having to continually ‘prove’ that they are cured, being perceived as being an ongoing source of infection, and living with the label of being infected with HCV.

P: Yeah. I’m going to live with it forever, even though I’m cured.

P: Yeah.

P: I’m still going to live with it, forever and ever and ever.

P: Yeah. I’m going to have it forever.

P: Yeah. All of us will.

P: Like, everybody that knows that I got, I had hep C, in their mind, I have it forever.

### Challenging stigma with population-based screening

Participants described a lack of visibility of hepatitis C in the public domain, with little information available to the general public regarding how the virus is acquired and, importantly, the availability of a cure. According to participants, this lack of public awareness allows stigma to persist and undermines efforts to eradicate hepatitis C. Moreover, participants recounted how the persistent conflation of hepatitis C with injection drug use and the manner in which such information can be managed by patients during health care encounters influences who gets tested and ultimately treated for hepatitis C.

P: If you go in there, and you’re middle class, and you look like you’re okay, they don’t assume that you might have something like hep C, whereby if somebody goes in there looking like a drug addict, they would assume that. And I spent many, many, many years, doing drugs, like, decades doing drugs. And not until I finally told the doctor, I, when I started using harm reduction, when I lost my home, and I went through university doing drugs; I went through working, you know, and all these years, and yet, no one knew. No one even thought, you know, that I was doing that. You know?

Given the limitations of a risk-based approach to screening and their own experiences of having no symptoms attributable to hepatitis C, participants described the importance of considering broader population-based screening strategies for identifying asymptomatic individuals otherwise perceived as being at minimal risk for hepatitis C. Notably, through their participation in the focus groups, participants identified older adults as being one potential group that could benefit from enhanced screening programs, an observation that is consistent with birth-cohort screening strategies endorsed as a means of identifying undiagnosed individuals [50, 51].

P: There may be a certain age group of people, I don’t know, that might be, I don’t know. But to me, it’s kind of interesting all of us are older.

Participants described several avenues to facilitate population-based screening, such as testing during annual physical examinations and integrating hepatitis C screening with existing breast and colorectal cancer screening programs [52, 53]. In addition to identifying and treating more individuals, participants speculated that moving beyond risk-based screening approaches could provide an opportunity to ‘normalize’ hepatitis C testing among the general public and begin to combat ignorance regarding the disease and its transmission.

P: Well, because, like, (name) was saying, the stigma. You know, I’m kind of in awe today, of that. A lot of people might have it and not even know it. So just a brochure that goes out in the mail, to every, whoever, that, that comes in the mail. You know, that there is free testing, and hepatitis seems to be growing. You know? That it’s important, just a flyer that it’s important to be tested through the Ministry of Health or somebody so that everybody gets a thing that, to bring up awareness that it’s not the stigma of drug addiction and all that. You know? And I’m sure just by being here today, there’s a ton of people out there that have it that don’t even know.

## Discussion

In our qualitative study, we found important differences in how HCV treatment was experienced by people who do and do not use drugs. Overall, our findings illustrate how structural stigma generated and reproduced through healthcare encounters limit access to DAAs among people who inject drugs.

Our findings have important implications for hepatitis C treatment delivery. Specifically, eliminating hepatitis C as a public health threat is predicated on equitable and low-threshold provision of DAA treatment to all individuals living with this condition [37, 54, 55]. However, our study demonstrates the multiple ways in which stigma operates to limit access to DAAs for people who inject drugs and thereby reproduce pre-existing health inequalities which impact this population. Most notably, our finding that interactions with healthcare providers are creating what past researchers have termed a ‘hierarchy of deservedness’ for DAA treatment, [56] further marginalizes and disempowers people who use drugs. These findings build upon past research demonstrating that health care provider assumptions and attitudes about the motivation and capacity of people who inject drugs to adhere to therapy and the possibility of reinfection act as barriers to treating this population, [57–59] despite evidence demonstrating that people who inject drugs are willing to undergo treatment and have DAA cure rates that are comparable to those of other populations [60–65]. Moreover, these findings are extensions of past debates regarding the futility of treating individuals with alcohol and substance use disorders with life-saving modalities such as liver transplants and cardiac valve replacements, and the deservedness of these individuals for such therapies [66–68]. Although participants described community-based and peer-supported HCV treatment programs as safe spaces in which to access HCV care, participants also described a haphazard approach to learning about these sites through others in their social circles, despite opportunities for referral or awareness of such treatment programs though multiple prior healthcare interactions. Processes that routinize referral to these programs at the time of diagnosis and through other settings commonly frequented by people who inject drugs (e.g., opioid agonist therapy dispensing pharmacy, drop-in centres) are needed to promote timely access to DAAs for these individuals. Furthermore, given the safety and simplicity of these drugs, expanding non-specialist models of care to allow delivery of HCV testing and treatment in settings such as shelters, substance use treatment programs and pharmacies is a promising approach for promoting low-barrier access to DAAs for people who inject drugs [69–73]. Research examining treatment outcomes and the acceptability of such models of care is needed. Involving people with lived experience in the development and delivery of programs is critical for diffusing power differentials, preventing stigma, and ensuring that all relevant supports are in place within such services [73–78].

In contrast to participants who inject drugs, participants who did not inject drugs did not endorse experiencing stigma though healthcare encounters. Instead, these participants generally recounted how interactions with healthcare staff provided respite from the felt stigma and identity disruption associated with hepatitis C. Further, referral to therapy was described as a logical extension of diagnosis, with extensive supports in place to facilitate adherence, follow-up testing and financial coverage for DAAs. Although one area of overlap between people who did and did not inject drugs was the embodiment of stigma as hygienic practices that would not be expected to interrupt HCV transmission, participants who did not inject drugs experienced ‘the cure’ as both virologic and social clearance, in that they felt liberated from the identity of the ‘dirty’ individual who is a peril to others. Conversely, this benefit was not perceived as attainable by people who inject drugs, who instead describe having their claims of being cured challenged by healthcare providers. This finding is an illustration of Goffman’s concept of ‘identity engulfment’, wherein people who inject drugs remain subject to expressions of labelling and stigma because hepatitis C remains central to how they are defined by others who have the power to do so, notably healthcare providers [42]. Moreover, the contrasts between people who do and do not inject drugs highlight Link and Phelan’s explication of stigma as a social process rooted in power and structural inequities, whereby access to DAAs and the opportunity to resist ‘identity engulfment’ are embedded within and influenced by prevailing discourses of health as an individual responsibility and intersecting forms of marginalization. Consequently, expanding non-specialist, low-threshold HCV treatment models alone is unlikely to provide equitable access to DAAs among all people with HCV. Rather, such programs must also attend to the many social processes and structural factors that reproduce stigma and exacerbate inequity for people who inject drugs [2–7]. Recent scholarship describing training in structural competence, where healthcare providers are taught to identify and attend to the social and political determinants of patient health, represents one promising area for integrating opportunities for structural change within novel hepatitis C treatment delivery programs [79, 80]. Participants also described the potential for population-based screening programs as ‘upstream’ interventions to normalize HCV testing and disrupt the potential for stigma produced with the current emphasis on risk-factor based screening. Although universal screening has recently been endorsed as a strategy for optimizing the diagnosis and treatment of hepatitis C, [81, 82] whether this approach can effectively counter stigma remains unknown.

Our study has some limitations. First, our sample includes only participants who were engaged in hepatitis C care, precluding us from making inferences about those with less engagement. However, we were interested in understanding the experiences of individuals who had received treatment and draw comparisons between people who did and did not inject drugs to explore the different dimensions of stigma and how these influence access to DAAs. Second, our study was conducted in a large urban centre, and may not reflect experiences of people with hepatitis C in rural and remote settings. However, our goal was to understand factors that limit DAA uptake in settings where hepatitis C treatment is otherwise not limited by the availability of specialists, treatment programs and harm reduction services. Third, we did not consider the perspectives of clinicians and policymakers. However, we elected to characterize the experiences of people with hepatitis C in the DAA era as few such studies have been conducted and the need for such research has been raised [73]. Finally, although our study provides some insight into the influence of social location on how hepatitis C treatment is accessed and experienced, we did not explore the manner in which various determinants of health (e.g., gender, race, health literacy, self-advocacy) intersect to produce stigma and inequality. Intersectional-informed scholarship on this topic is therefore an avenue for future research.

In conclusion, our work demonstrates how various dimensions of hepatitis C related stigma limit access to curative DAA therapy for people who inject drugs. Developing and ensuring unfettered access to novel HCV treatment delivery programs that attend to the social and structural determinants of stigma are needed to facilitate further scale up of DAAs in this population and support goals of eradicating hepatitis C as a public health threat.

## Data Availability

The datasets generated and analyzed during the current study are not publicly because they contain information that could compromise participant privacy and consent.

## List of abbreviations

HCV: Hepatitis C viral
DAAs: Direct-acting antivirals
REB: Research ethics board
