# Correction: Accessing hepatitis C direct acting antivirals among people living with hepatitis C: a qualitative study

**DOI:** 10.1186/s12939-023-02003-4

**Published:** 2023-10-11

**Authors:** Tony Antoniou, Cheryl Pritlove, Dana Shearer, Mina Tadrous, Hemant Shah, Tara Gomes

**Affiliations:** 1 Li Ka Shing Knowledge Institute, Unity Health, Toronto, ON Canada; 2 Department of Family and Community Medicine, Unity Health, Toronto, ON Canada; 3 Department of Family and Community Medicine, University of Toronto, Toronto, ON Canada; 4 Dalla Lana School of Public Health, University of Toronto, Toronto, ON Canada; 5 Leslie Dan Faculty of Pharmacy, University of Toronto, Toronto, ON Canada; 6 Women’s College Research Institute, Toronto, ON Canada; 7 Toronto Centre for Liver Disease, University Health Network, Toronto, ON Canada; 8 Department of Medicine, University of Toronto, Toronto, ON Canada; 9 Institute for Health Policy, Management and Evaluation, University of Toronto, Toronto, ON Canada

**International Journal for Equity in Health (2023) 22:112**

10.1186/s12939-023-01924-4

After publication of this article [1], the authors reported that in the Sampling and Recruitment section, the phrase ‘We partnered with an outpatient specialist-led liver clinic affiliated with a large teaching hospital and three clinics of the downtown Toronto Community Hepatitis C Program to assist us in recruiting a diverse sample of participants according to sex, age and current or past history of injection drug use. The Toronto Community Hepatitis C Program provides low-threshold access to DAAs and additional support for people who use drugs or alcohol [39].’ should have read ‘We partnered with an outpatient specialist-led liver clinic affiliated with a large teaching hospital and three primary-care based hepatitis C clinics to assist us in recruiting a diverse sample of participants according to sex, age and current or past history of injection drug use.’

As part of this change, reference 39 has been removed.

The original article [1] has been corrected.
